# Conducting in-depth interviews with and without voice recorders: a comparative analysis

**DOI:** 10.1177/1468794119884806

**Published:** 2019-11-07

**Authors:** Rwamahe Rutakumwa, Joseph Okello Mugisha, Sarah Bernays, Elizabeth Kabunga, Grace Tumwekwase, Martin Mbonye, Janet Seeley

**Affiliations:** MRC/UVRI and LSHTM Uganda Research Unit, Uganda; MRC/UVRI and LSHTM Uganda Research Unit, Uganda; School of Public Health, University of Sydney, Australia; Department of Global Health and Development, London School of Hygiene and Tropical Medicine, UK; MRC/UVRI and LSHTM Uganda Research Unit, Uganda; MRC/UVRI and LSHTM Uganda Research Unit, Uganda; MRC/UVRI and LSHTM Uganda Research Unit, Uganda; Child Health and Development Centre, Makerere University, Uganda; MRC/UVRI and LSHTM Uganda Research Unit, Uganda; Department of Global Health and Development, London School of Hygiene and Tropical Medicine, UK

**Keywords:** Data collection, interviews, group discussions, audio recording, field notes, transcription, trustworthiness, rigour

## Abstract

The use of audio recordings has become a taken-for-granted approach to generating transcripts of in-depth interviewing and group discussions. In this paper we begin by describing circumstances where the use of a recorder is not, or may not be, possible, before sharing our comparative analysis of audio-recorded transcriptions and interview scripts made from notes taken during the interview (by experienced, well-trained interviewers). Our comparison shows that the data quality between audio-recorded transcripts and interview scripts written directly after the interview were comparable in the detail captured. The structures of the transcript and script were usually different because in the interview scripts, topics and ideas were grouped, rather than being in the more scattered order of the conversation in the transcripts. We suggest that in some circumstances not recording is the best approach, not ‘second best’.

## Introduction

The use of audio recordings has over the past 30 years become a taken-for-granted approach to generating transcripts of in-depth interviewing and group discussions (Lee, 2004: 878), their use a ‘normalised discursive practice’ (Nordstrom, 2015: 388). Tuckett (2005: 33) suggests that the use of a recorder is necessary to ‘counter criticism’ that qualitative research is, quoting May (1991: 190), ‘prone to systematic bias’. Seale and Silverman (1997) list among the strategies required to ensure ‘rigour and validity’ in qualitative research: ‘Recording data objectively and comprehensively, including the use of audiotapes, videotapes and different levels of details in the transcription of data’ (p. 380).

Rapley (2004) discusses the influence a recording device may have on an interview, concluding that the recorder is just one of many influences on the interaction and any untoward influence may be balanced against the benefit of recording the encounter, once appropriate assurances about the use of the recording have been made, and allowance made for things the interviewee may wish to say ‘off tape’. Nordstrom (2015: 389), however, argues that ‘recording devices – social science tools or apparatuses – are not mute or innocent entities that simply record interviews’; the recording devices have a place in the interview and have an influence on the data. In situating her discussion of the place of recording devices in interview settings, she summarises the literature which documents the normalisation of recording devices as a tool to ‘capture data’ that are ‘apolitical, acultural and aproblematic’ (p. 390). Nordstrom goes on to note that recording devices ‘can be linked to race, age, class, culture, and politics even though the qualitative inquiry and anthropological literature many times do not explicitly acknowledge these areas’ (p. 391). The influence of a recording device may remain in situations where the recorder is turned off: the very existence of the device, and the intention of the interviewer to record, even if the recorder is put out of sight can mean that it still has a presence in and an influence on the interaction.

The assumption that interviews are audio recorded can result in authors providing very limited information on the circumstances of the data collection, beyond that recordings were transcribed. Yet, the expectation that audio recorders should be used can, in our experience, result in journal reviewers requiring additional information on the quality of the data collected (in 2015 a reviewer of one of our papers observed, ‘Data recording [p. 4, lines 3-7]: the authors should provide some assurance that the notes taken were accurate, given the lack of audio recording’) or a request to note the lack of the use of an audio recorder as a limitation in the paper.

In this paper we begin by describing circumstances where the use of a recorder is not, or may not, be possible; we then go on to share our comparative analysis of recording transcriptions and scripts of interviews made from notes taken during the interview. We show that the data quality between audio-recorded transcripts and interview scripts written directly after the interview were comparable in the detail captured. In our discussion we highlight the importance of training for researchers conducting interviews not only to ensure data quality but also to be responsive to contexts where using a recorder is not appropriate.

Our purpose in writing this paper is not to discount the value of recordings (when used appropriately) but to reprise Nordstrom’s words of caution over the place of the recording within the interview. We suggest that in some circumstances *not* recording is the best approach, not ‘second best’.

### When recorders have no place

In 2008 we, social scientists with the MRC/UVRI and LSHTM Uganda Research Unit, began qualitative data collection with women at high risk of HIV infection in Kampala, Uganda. The women had been recruited into a dedicated clinic serving an epidemiological cohort for the study of HIV and other sexually transmitted infections and the design of appropriate prevention and treatment interventions. Our purpose was to study the social context of sexual partnerships among the women to explore the nature of sexual partnerships, risk perceptions and risk behaviour. Many of the women sold sex to earn a living. Sex work is illegal in Uganda. In consultation with the women being recruited into the cohort, it was agreed that photographs would not be used as a form of identification at the clinic and that recorders would not be used to ‘capture their voices’. More than ten years on we do, for some social science research studies, use recorders (for group discussions), because trust has been built that we will not misuse their voices, but overall because of many women’s nervousness over their voices being recorded, we write up interview scripts from notes (see, for example, Mbonye et al., 2013; Rutakumwa et al., 2015; Schulkind et al., 2016). This approach is possible because we have a very experienced team of social scientists with excellent interviewing and documentation skills. We also take care to ensure that we treat the women’s consent to participate in interviews as a process, whereby we reprise the terms of our ‘contract’: what we have agreed with participants about the data collection and use each time we meet. We also share the study findings (not individual details) with study participants for their comment as well as information. Conducting an interview, with or without a recorder, can appear to be something anyone can do, but like participant observation (which also can seem to non-specialists deceptively simple, (see, May, 2001: 153 ff)), the art of interviewing and documenting the encounter afterwards is demanding.

Another setting in which we rarely record interviews is at fishing sites, when interviewing both men who work in the fishing industry and women who may trade in fish or work in the service sector at the fishing sites (for example, bar work, restaurant work and sex work). Given the topics of conversation, often related to sexual or occupational risk (including illegal fishing activities), the preference of respondents has been not to be recorded (see, for example, Pearson et al., 2013).

Sometimes, as Rapley (2004: 19) notes, it is appropriate to turn the recorder off during an interview at the interviewee’s request because something is ‘confidential’ and she/he do not wish her/his voice to be captured relaying the information. Sometimes, the decision to turn off the recorder comes from an interviewer aware that the presence of the instrument makes an interviewee nervous. Nordstrom (2015: 392) recounts such an instance where initially she tried hiding the recorder from view to put the interviewee at ease which had the desired effect until the interviewee requested that the recorder be turned off.

Shaver (2005) calls for a ‘participant-centred approach’ in her paper, setting out guidelines for ‘ethical, non-exploitative methodologies’ for working with hidden populations such as sex workers. Some of the techniques she advocates for data collection to build trust, such as ‘working the stroll [the recognized area on the streets for soliciting]’ which involved spending time observing and being observed by sex workers and engaging in conversation, would preclude the use of a recorder. The overt use of a recorder would affect the informality of the process; any covert use would abuse the trust being built. We have used similar informal walking methods (Bond et al., 2019) as part of our own work in fishing sites and in trading centres not only to learn about the place and refine our research questions but also in order to be seen and be known.

Glaser (1998: 107; 2002) refers to the worrisome accuracy attributed to taping interviews, in his discussion of the use of interviews in grounded theory. He suggests that recording interviews focuses attention on the interview data and not the broader experience of the research which includes observation and personal interactions. Stern (2007: 119) cites Glaser’s work and practice of grounded theory and suggests that ‘researchers [using a grounded theory approach] need to focus on the accuracy of their discovered truth, rather than the less important what-did-they-say-exactly’. The assumption that information from any interview, whatever method of data collection is used, is ‘accurate’ ignores the fact that interviewees may provide inaccurate information to protect their privacy or they may tell you what they think you want to hear (Shaver, 2005). Deciding about whether to record or not, or whether to turn a recorder off mid-interview, can influence the decisions interviewees take about the information they share.

We are not, we would stress, suggesting that using recorders is necessarily ‘bad’ research practice; instead, we suggest that there should be a choice of method, with the most appropriate approach being used considering the contextual factors which influence data collection, as would apply to any choice in the application of qualitative methods.

That said, poorly documented interviews drawn from notes will result in poor-quality data. All interviewers, whether they use a recorder or not, require careful training.

In the next section we report findings from a comparative study of recordings and interviewer notes/memory. Using primary data from a recently concluded study, we examine the content of scripts (we use this term to denote the product of the interview prepared from notes) and verbatim interview transcripts to compare the data captured using both methods.

## Methods

We conducted a sub-study within a study that was examining the well-being of older people on antiretroviral therapy (HIV treatment) in a rural setting in Uganda to explore the differences between data produced from audio recordings and those produced from notes and memory of the interview. In this study – which was led by one of the authors of this paper, JM – data were collected by two trained and experienced interviewers (both with more than 20 years of experience of qualitative data collection) (co-authors EK and GT) who used voice recorders as a data capture method. They conducted 30 interviews each. All interviews were conducted in Luganda, the main local language. As part of our sub-study, the interviewers, who were also trained and experienced in data capture without the use of voice recorders, were asked to prepare scripts in English of some of the interviews they had conducted *without* listening to the voice recordings. Preparation of these scripts was done immediately after each interview, preferably the same day, to minimise recall bias. Scheduling time for this activity was important for data management. The voice-recorded interviews were transcribed verbatim and translated into English by someone other than the interviewer. For this paper, 60 pairs of scripts/transcripts (each pair consisting of the transcript from the recorded interview and the script from the notes of the interview) were analysed and compared. The initial comparison was conducted by EK and GT. Subsequently four other co-authors of this paper (RR, JM, MM and JS) conducted further analysis and cross-checking to verify the initial findings.

## Findings

### Comparing content in the verbatim transcript and the detailed script

Verbatim transcripts were in all cases more detailed than scripts, with transcripts on average being double the length of scripts. Nonetheless, the reason for this difference was often because of the inclusion of what Stern (2007: 118) calls ‘filler’ (content not directly on the topic of the interview, which can be summarised in a brief account in the script). We found that the content of the scripts was generally comparable to that of verbatim transcripts in terms of how it depicted the key themes in the interview. To illustrate this point, we review extracts of coding from verbatim transcripts and those from their corresponding scripts. Our focus was specifically on four codes: *general health status; experiences of isolation and its negative impact; treatment adherence*; and *experiences of HIV diagnosis*.

With respect to the code *general health status*, the following is an excerpt from a verbatim transcript of an interview with a 65-year-old man:

Interviewer: *How can you rate your quality of life nowadays?*

Respondent: *My quality of life is good because I do not suffer from malaria, flu and cough. People from Virus [MRC/UVRI] tested my blood but they did not find any fever [malaria]. I take long to get fever and what has helped me from not falling sick is the glass of water I take every morning after waking up. [However] the accident that I got has made me feel a burning sensation on my spinal cord . . .. That is the only problem I have . . . it requires me to go for surgery.*

Interviewer: *Surgery for what?*

Respondent: *For the spinal cord. I got a cervical injury, so it requires me to go for surgery.*

Interviewer: *How is your mental health?*

Respondent: *Am very settled. Even now I feel am settled because I do not have debts . . . I do not have any problem. I can get sleep at any time I feel like. Even yesterday, I switched off my television at around 8:45 pm and went to sleep. I woke up at 2:45 am and slept again till I woke up at 6 am.*

Interviewer: *At what percentage can you rate your quality of life . . .?*

Respondent: *70 percent.*

Interviewer: *Why 70 percent?*

Respondent: *It is because I rarely fall sick, and I have never fallen sick to the extent of being admitted to a hospital. If it was not because of the accident, my [quality of] life would have been 90 percent.*

Looking at the above excerpt, we note that the participant touches on three key issues: his self-perceived quality of life (about physical health), his mental health and a strategy to promote good health. The following is the corresponding excerpt from the script:

When asked how he feels, he replied that he is okay because he does not suffer from malaria or headache. He added that even people of MRC bled him and when they examined his blood, they did not find there anything. He said when he wakes up in the morning he drinks a glass of cold water before starting his activities [and that this] was something that keeps him healthy.

About his mental health, he said he is settled because he does not have debts, and in case of treatment fees his daughter clears those. He says he sleeps well and generally he has no problem.

I asked him about the symptoms or any illness he experienced in the last 30 days, and he responded that he never experienced any sickness. But that once in a while when he feels unwell he goes to the health centre [although he adds] it is very rare.

When I asked what things affect his daily wellbeing, he replied that [this may happen if] he wanted to eat good sauce such as fish and he fails to get it because it is very expensive. He rated his quality of life to be around 70 percent because he does not fall sick from time to time, except the pain from the accident he got while he was still living in Kampala. If it was not for that pain, he said he would rate himself at 100 percent.

The three key issues highlighted in the transcript are not just restated in the script but also presented in their complexity, that is, they are described to the extent that virtually all the different aspects of the issue are captured. For example, in portraying the participant’s statements on his mental health, the detailed script echoes the verbatim transcript by highlighting not only the participant’s self-assessment (a settled mind) but also how it manifests (good sleep) and what he attributes his mental health status to (no debts). Likewise, the script highlights not just the participant’s perception of his quality of life as being good but also the reasons why he arrives at this assessment (does not suffer from malaria or other common ailments). Of note, the transcript records the participant saying that he would feel 90% without the pain, while the script gives 100%.

Another code was experiences of isolation and its negative impact. An interview with a 69-year-old man:

Interviewer: *Do you have any way you are affected by the physical care you receive at home?*

Respondent: *I do not have enough physical care because the grandchildren do their own things and I also do my own. I am the one who takes care of myself*.

Interviewer: *Does it mean that you cook and wash for yourself?*

Respondent: *I cook for myself. It is so tiresome coming back from digging and you go back and cook for yourself at home. A family needs to be led by two people for sure!*

Interviewer: *In the research which was carried out in the past [there has been other research with older people in this setting, which some of those interviewed during this study had been involved in], it showed that older people feel socially isolated. Has such a thing ever happened to your life?*

Respondent: *Yes it usually happens to me. I can spend a full week without having food to eat and [I have] no radio to listen to in order to know what happens in the world.*

The participant’s discussion of experiences of isolation and its negative impact revolved around two key unmet needs: physical care and being informed about what is happening beyond his community. In the corresponding script below, both issues are covered.

He [respondent] explained that he did not receive enough physical care because the grandchildren can do little – they are still young and may not be as responsible as an adult person would be.

Asked if he meant he washed for himself and did the cooking for example, he explained he did and that they (those domestic activities) are also tiresome being that he does them after digging.

Asked if he has ever felt socially isolated, he explained that it happens like when he takes a full week having failed to get what to eat, even failure to have a radio and he feels lonely/left out without even knowing about what is happening in the country.

While from the transcript we learn that the grandchildren are ‘doing their own things’, it is only in the script that we learn that they are young and not able to provide care; this information is based on the interviewer’s observation at the home.

There is one inconsistency between the transcript and the script: the use of *country* instead of *world* in the script. This was an issue of translation: the participant used the word ‘ensi’ which can be used to mean both country and world.

For the coding for *treatment adherence*, a 60-year-old woman discussed the challenges she encountered:

Respondent: *I take them [drugs] in the morning and the health workers told us to swallow the drugs after you have taken something [food]. The thing is that I feel so bad lacking food to eat yet I have medicine to take on a daily basis, so that is why I sometimes miss taking my drugs.*

Interviewer: *How often have you missed taking your medicine due to lack of food?*

Respondent: *They are so many times. One day I took those drugs on an empty stomach but I was almost dying, I felt like I was drunk! When you take medicine after having eaten something, you feel better than taking it on an empty stomach. That medicine is very strong and it needs to be swallowed after having eaten something . . . . Therefore I have to make sure I take my medicine every day but after eating something and when I fail to get food, I do not take the medicine.*

Interviewer: *How do you make sure you take your drugs at the right time and when required like the health workers told you?*

Respondent: *I have nothing which reminds me to take my drugs. It became my consciousness to take the medicine the way they directed me to take them. I grasped it that the moment I wake up like this in the morning, I have to take my medicine.*

It was clear from this participant’s perspective that food was a critical aspect in treatment adherence: highlighted in the transcript were issues such as the adverse event after taking the drugs on an empty stomach, the resolve not to take the drug without food and the high frequency of skipped doses due to lack of food. The script captures these elements:

The older woman continued explaining: ‘they [health workers] tell you; ‘you have to swallow the drugs after you have taken something [food]. However, there are times when I do not swallow the drugs having failed to get what to eat and I become confused’, Narrating about the challenges faced since she started taking her drugs, the older woman said; ‘failure to have food’.

Asked how often she has had this challenge, she said it has happened very many times. She continued; ‘I at one time tried to swallow those tablets when I had not eaten anything and I felt as if I had become drunk! The drugs are very strong and I almost collapsed. . . .’

Narrating about the instructions she got about her HIV medicine, she explained; ‘you must first eat then take the drugs. You do not have to miss taking it’, She continued explaining that when one has no food/eats, taking the drugs becomes very challenging!’

As far as how she makes sure she has taken her HIV medication on time and when required, she quickly said; ‘my heart fully grasped it; immediately I wake up, I go and get my drugs then swallow. The only challenge comes in as I have already explained to you; when I do not have food, I do not attempt swallowing the drugs! I fear its strength’,

With regard to the theme *experiences of HIV diagnosis*, below is an account from a 57-year-old man:

Interviewer: *Were you suspicious that you would be HIV positive before you went to test yourself?*

Respondent: *I got attacked by monotonous fever. I could get attacked by fever all the time and I could feel cold all the time. Therefore, falling sick all the time made me to become suspicious that I may be HIV positive. The good thing [is that] my wife was so brave and encouraged me to go and test for HIV. If she had not encouraged me, I wouldn’t have gone for HIV testing, so when I came back home, I told her that I was tested positive and started on Septrin [cotrimoxazole]. That was around 2012, my record book is even here*.

Interviewer: *Which health centre did you go to?*

Respondent: *I went to xxx health centre. By then my CD4 count was around 560 but when I went back, they had reduced to around 370 or 400, so they told me that I am supposed to be started on antiretroviral therapy (ART). I felt so scared starting ART because I heard people saying that when you start on ART, you take it for a life time. I had a feeling that what if the drugs get out of stock, will I not die soon? The health worker encouraged me and told me that the medicine can’t get finished afterwards she told me that it would be better I take my wife there so that we can both be started on the drugs*. *When I went and told my wife, she also accepted but surprisingly, our CD4 counts were the same. We were given Septrin until time reached and they started us on ART. I started first ART for round two to three months and later they told me that my wife should also start. My wife also first got scared to start ART but the health workers encouraged me to advise her to allow starting ART since she may get attacked by TB so I also accepted to be my wife’s counsellor and I advised her to start taking the drugs. After talking to her, she accepted to take the drugs*.

In this excerpt the participant had highlighted his recurrent sickness that made him suspicious that he might be HIV positive and the important role his wife played in encouraging him to take the test; how he got to know about his HIV-positive status and the facility at which he was diagnosed; being started initially on cotrimoxazole; and getting his wife to accept an HIV test that turned out to be positive. In the corresponding excerpt below, these issues are covered.

Exploring how he came to know he is HIV positive, he told me that he suffered serious malaria; he bought tablets from the shop and swallowed them. The following day his condition had worsened (he had developed a mental problem and he was unconscious). He was rushed to xxx sub-dispensary for treatment. At the facility he was put on a drip but since the condition was not improving at all they referred him. While his people were planning to take him away from the facility one nurse who he taught in school came across them and advised them that she has observed the patient’s condition, she will manage to treat him from home. They transported him back home and this musawo [health worker] started offering treatment from his home. He recovered after two weeks. He told me that after recovery his wife advised him to go and test for HIV at xxx health centre. He said that he went for an HIV test and it’s where he was informed that he was HIV positive. When he returned home he informed his wife about it because she was the one that encouraged him to go for the test.

Probing if he was suspecting anything before going for an HIV test, he said he had experienced persistent fever. He said when he tested HIV positive right away they initiated him on septrin. He narrated that since his results were HIV positive abasawo [health workers] advised him to bring his wife for an HIV test as well. She went for an HIV test and she was also positive. She got shocked about what they were going to do but never misbehaved such as abusing him. He said when they tested his CD4 count it showed he had 500 cell. Even his wife’s CD4 count was in the same range. She was also started on septrin. He told me that he started ART when he had a CD4 count of 360 cells. He was the first to be initiated on ART in 2013 followed by his wife.

There are some small variations in the figures quoted for the CD4 counts, but in general the script is consistent with the transcript in highlighting the key point that the participant’s CD4 count had reduced at the time he was initiated on ART.

In one case the material coded from the script was not reflected in the corresponding verbatim transcript because the recorder had been turned off at the participant’s request. This involved an 80-year-old woman. The script included the following information:

She further explained that her newly married daughter-in-law; a third wife to her second born son cares for very much when she goes there. She washes for her, provides tea for her 4 times. This woman (daughter-in-law) works as a cashier for the maize mill of her [participant’s] son. [Participant said] she is very caring. However, what hurts her is using the same toilets inside her children’s house! Secondly she could not imagine if she died while there; how her son there could transport her dead body back. Using the same toilets with her grown up children hurts her because according to the Baganda culture, it is not acceptable for a parent to urinate or have long calls in toilets under same roof of her grown up children’s residence. They associate it with causing “obuk”, – a disease suspected to attack an older person making her shake all the time.

The participant was uncomfortable with sharing toilets with her adult children because of the belief that such sharing causes a traditional disease and had not wanted her voice recorded talking about this issue.

1. *Comparing and examining the structure of transcripts and summaries*

A relevant but unanticipated finding from our comparative analysis of verbatim transcripts and scripts relates to structure. Ideally, the verbatim transcript is a word-for-word written and translated version of the interview, usually including repetitions and fillers but also a record of non-verbal events such as expressions of emotion (which the interviewer records in the field notes which should accompany the transcript to provide information on the conduct of the interview). On the other hand, the script is the fieldworker’s representation of the interview dialogue to the extent that memory and notes can allow, but typically featuring at least key issues/themes emerging from the interview dialogue.

In comparing the structure of verbatim transcripts with that of the scripts, we noted that the narrative in the summaries did not follow the order in which the questions were asked and that as a result, issues that came first in the verbatim transcript sometimes appeared later in the script (or vice versa). As a result, the script represents a first stage of analysis and sorting by reordering and grouping material. This practice was a conscious effort by the interviewer to synthesise all aspects of the data that speak to a given issue.

To illustrate this point, we present material from the interview of a 60-year-old woman’s perspectives on HIV disclosure. The verbatim transcript contained the following information in one section:

Interviewer: *Do you have any person you disclosed your status to?*

Respondent: *May be my sister*.

Interviewer: *Why did you decide to disclose to her?*

Respondent: *Don’t you know that when I fall sick she comes here and takes care of me! When she also falls sick, I take care of her. I also disclosed to her like I have told you that “they draw our blood samples but they do not tell us our results”*,

Interviewer: *You mean you were not given any medical form?*

Respondent: *No*.

Interviewer: *How did your sister feel after disclosing to her your status?*

Respondent: *She did not feel bad*.

Interviewer: *Which advice did she give you?*

Respondent: *She encouraged me to go to the hospital and I start on the medication. If you are sick and you fail to take medicine, you die very soon but when you start on the drugs, you can live longer*.

By comparison, the corresponding script included additional material related to *disclosure* in addition to the information about the sister contained above:

Regarding whom [participant] has ever disclosed her status to, she said it was only her sister who lives elsewhere in the same village that she informed. It is because she is the one she expects to nurse and care for her when she fell sick. She continued explaining that she still informed her sister like she had informed me plainly but not having any document from the health workers which would prove to anyone that she was tested and found HIV positive.

Probed about her son with whom she stays in the house, the older woman said she did not disclose to him because she feared to scare him. She instead told him about her liver results and the son knows that the treatment she takes daily is for her liver problem. She did the same to her other children, telling them she is taking medication because she was diagnosed with liver problems.

When she disclosed to her sister, she [sister] encouraged her to go to the health facility and be given the drugs because failure to take the drugs would make her health weak and eventually die and that once started on to the drugs, she should take for the whole of her life because stopping to take it for some time once started, her health would be ruined.

Reviewing the verbatim transcript, we noted that the interviewer had drawn several bits of data from different sections of the interview into the material on disclosure, effectively assembling relevant but scattered aspects of disclosure into one section. For example, the participant’s disclosure of her HIV-positive status to her sister appears on page 14 of the verbatim transcript; non-disclosure to her son is reflected on page 13 of the transcript; and revealing to the son and his siblings that her daily medication was for a liver problem in order not to scare them is featured on page 18 of the transcript.

In another case we reviewed the coding of *stigma* from an interview with a 64-year-old woman. The verbatim transcript featured the following data:

Interviewer: *Have you ever felt stigma because of being HIV positive?*

Respondent: *At first you may feel fear but afterwards you gain strength/courage*.

Interviewer: *What makes you gain strength?*

Respondent: *Your friends can make you gain courage. I may see a child of 15 or 18 years dying of HIV/AIDS and I wonder a person who is 60 years old like me not to get infected! For sure God has protected me so that is why my heart became strong*.

Interviewer: *Do you not fear people seeing you taking your tablets?*

Respondent: *Do you mean Septrin?*

Interviewer: *Yes!*

Respondent: *No, it does not scare me. They even explained to us and they told us that Septrin helps to fight illnesses which may attack a person who is HIV positive. They told us that it does not treat HIV/AIDS but it prevents other illnesses from attacking you*.

We then compared this with the excerpt from the script:

She reported that if you lose a partner even if she died of something else they [people] comment associating his death to HIV/AIDS. She said that when you inform people/neighbours about personal issues they just laugh at you, but they do not support you in any way.

Probed if she suspected her husband to have died of HIV, she narrated that she knew only that she went for HIV test to confirm.

I went ahead and asked her if she ever experienced stigma because of being HIV positive. She answered that at first she felt stigma but later got firm because of other people who had the same problem. She said you see children who have passed away having been claimed by HIV at 15 years old, and then those who die at 60 years old. She said if she dies at her age, why does she not thank God for protecting her!

Consistent with our observations from the earlier case, the script includes material from elsewhere in the interview on the same topic.

## Discussion and conclusion

In this paper we are interrogating the increasing acceptance that audio recording interviews is singularly best practice and considering what might be equivalent, lost or gained if we rely on highly trained interviewers to write interview scripts from brief notes and their memory. The comparison of the data quality between audio-recorded transcripts and interview scripts written directly after the interview indicated that they were comparable in the detail captured. However, we observed that some of the filler material was edited out in the interview script and this could lead to the omission of some valuable detail, particularly if an interviewer knows the context well and takes certain things for granted as ‘known’, which would appear in the transcript of an audio recording. The structures were different with the interview scripts being generally grouped into topics and ideas rather than the more scattered order of talk in the transcripts.

This study emphasises what is consistently found but often dismissed which is the importance of training and the quality of the researchers; this more than anything will affect the quality of the interviews and data collected. If interviewers are not sufficiently trained to understand the effects of their approach, questions, tone and responsiveness within the interview on the quality and pertinence of the data collected, then whether one uses audio recorders or not it will produce poor data. An interviewer should be trained to read and respond to the interview encounter to minimise any discomfort and support the participant’s confidence in the value of the contribution of the account (Tuckett, 2005). This should involve being reflexive about the research relationship (the differences and similarities between interviewer and interviewee) and responsive to the variant discursive contexts provoked by the interviewer’s enquiries (Hewitt, 2007; Karnieli-Miller et al., 2009). Prior experience of and skills in qualitative analysis are also extremely valuable in enhancing the quality of interview scripts.

We are accustomed to there being a point in an interview which warrants turning the recorder off. However, a situational recognition of the powerful impact of an audio recorder in an interview which may be shaped by the dynamics of the interviewing dyad, the nature of the interview questions and/or the broader context, may require the researcher to not turn the audio recorder on or never intend to use one at all. As with all aspects of our choice of methods as qualitative researchers, we must attend to the influential specificity of context. Choosing not to use an audio recorder, because of a likely negative impact, when the interviewer is sufficiently trained to produce interview scripts should not be viewed as a weakening of research conduct but rather as a successful indicator of the researcher’s sensitivity to the integrity of the research project.

The training needs to extend to equipping interviewers with a nuanced and flexible understanding about which data are likely to have relevance to the research questions. This has direct implications for the quality of the interview scripts. A trained interviewer can discern any aspects of small talk that may have a connection, however tangential, with the research topic and so include them in the script. Such training requires an investment of time and supervision. As part of the training process where we have used interview scripts in preference to recorders in other studies conducted by the authors (SB and JS), practise interviews have been audio recorded and interviewers asked to write up the interview scripts without referring to the audio recording. They have then later transcribed the audio recording of the same interview. With their supervisors, they compared these two versions and, where appropriate, areas of additional detail were identified for inclusion in the scripts. Once we were confident in the quality and fullness of the practise interview scripts, the interviewers were ready to commence the study data collection without audio recording the interviews.

As noted above, the structure of the scripts, in which details of the interview account were grouped into topics, reflects not only the topics covered in the interview guides but also those raised but unanticipated, which are of interest to the researcher. This indicates the researcher’s capacity to conduct a very preliminary analysis of the data collected, which can immediately inform subsequent interviews, and facilitates iterative data collection and analysis in real time.

There is a considerable literature which takes for granted the notion that audio recording interviews inevitably produces more accurate and therefore better interviews (Green and Thorogood, 2009: 101ff.; Lee, 2004; Paulus et al., 2017; Tuckett, 2005). The intellectual logic underpinning this is that audio-recorded data, which constitute the ‘facts’ of the interview account, will produce more valid and trustworthy data. This is an indurate argument that risks erring into the confining parameters of trustworthiness which apes the consistency of quantitative research and does not capitalise on the contextual depth and responsiveness offered by qualitative research. It is predicated on the assumption that the path to accuracy is narrowly followed by capturing precisely what is said, rather than the purpose of an effective interview which is in part about enabling an environment in which participants feel comfortable to say what they want about a particular topic (Oakley, 2016).

Presuming that audio recording an interview will always produce more ‘accurate’ data ignores the influential effect of specific contexts on what can be said about a particular experience and what can be ‘trusted’ in what is said. It also neglects the influence of the recording device on what is said and done (Caronia, 2014). Rapley (2004) argues that this can be countered by including field notes which recount what is said once the recorder has been turned off. However, this illuminates the demonstrable political effect of the audio recorder in that what is said once the recorder is turned off is often acknowledged to be particularly revealing about a topic (Nordstrom, 2015). It is also worth noting how infrequently field notes are presented, alongside transcript extracts, in peer-reviewed publications as important textual records of the influence and effects of context. The emphasis on audio recording pushes the singularity of the transcript as the ‘valid’ data source. The risk is that we lose what falls off the transcript (Sandelowski, 2002). Worse still is that we might not notice this omission because the effect of the presence of the audio recorder, whether turned on or off, is such that the participant’s circumspection means that something might not get said at all. There is a need to ‘develop a reflexive awareness of how recording methods affect the research process’ (Lee, 2004: 882).

Interviews are commonly characterised as an exchange or as a ‘gift’, an exchange in that the data are unavoidably a product of the encounter between the interviewer and the interviewee and a gift in that the interviewer commonly leaves with the data. However, as Oakley argues an essential question of conditionality as it applies to research is ‘whether the researched agree to take part on the understanding that they will not be given, in return, the chance to control the research product’ (2016: 208). To consider the production of interview data as a ‘gift’ is to also accept the inescapably unequal power inherent in the relationship between interviewer and participant. This may be made even more stark if the participant is discussing experiences which they may find shameful or incriminating, such as sex work or illegal fishing activities as in our own work. The uneven distribution of power may be concretised by the role of the audio recorder, in which the spoken word becomes indelibly recorded and taken away to be used as the researcher sees fit. This may curtail not only what answers participants may give but also what questions an interviewer may ask. What happens to the audio recording and transcript engages with ethical issues of trust and reciprocity, in which the researcher should act with integrity so that no consequent harm befalls the interview participant. However, an additional counterbalance employed by participants may be to not risk disclosing information that could inadvertently be used against them (Collins, 1998). This may produce accounts in which the participants self-consciously edit their narrative to provide a partial explanation of their experience.

Although characterised as a neutral and indispensable tool of qualitative interview research, the audio recorder is highly political. Without it, the research community may deem its absence a threat to the apparent validity and credibility of the data. Yet, using it may enhance both the potential vulnerability and circumspect nature of data produced through the interview encounter.

In their paper outlining the ‘Consolidated criteria for reporting qualitative research (COREQ)’ Alison Tong et al. (2007) observe that ‘Generally, audio recording and transcription more accurately reflect the participants’ views than contemporaneous research notes [. . .] Reasons for not audio-recording should be provided’ (p. 356). The COREQ checklist is now included in the submission guidance offered by publishers of journals which include qualitative research (see, for example: http://cdn.elsevier.com/promis_misc/ISSM_COREQ_Checklist.pdf). We would suggest a revision to the COREQ checklist to allow for the reasons *for* using an audio recording *rather than just ‘not audio-recording’*, to be provided.
